# Viral diversity and host associations in microbial electrolysis cells

**DOI:** 10.1093/ismeco/ycae143

**Published:** 2024-11-15

**Authors:** Marie Abadikhah, Frank Persson, Anne Farewell, Britt-Marie Wilén, Oskar Modin

**Affiliations:** Division of Water Environment Technology, Department of Architecture and Civil Engineering, Chalmers University of Technology, Sven Hultins gata 6, SE-412 96 Gothenburg, Sweden; Division of Water Environment Technology, Department of Architecture and Civil Engineering, Chalmers University of Technology, Sven Hultins gata 6, SE-412 96 Gothenburg, Sweden; Department of Chemistry and Molecular Biology, University of Gothenburg, SE-405 30 Gothenburg, Sweden; Division of Water Environment Technology, Department of Architecture and Civil Engineering, Chalmers University of Technology, Sven Hultins gata 6, SE-412 96 Gothenburg, Sweden; Division of Water Environment Technology, Department of Architecture and Civil Engineering, Chalmers University of Technology, Sven Hultins gata 6, SE-412 96 Gothenburg, Sweden

**Keywords:** bioelectrochemical systems, metaviromics, metagenomics, microbial electrolysis, phage-host associations

## Abstract

In microbial electrolysis cells (MECs), microbial communities catalyze conversions between dissolved organic compounds, electrical energy, and energy carriers such as hydrogen and methane. Bacteria and archaea, which catalyze reactions on the anode and cathode of MECs, interact with phages; however, phage communities have previously not been examined in MECs. In this study, we used metagenomic sequencing to study prokaryotes and phages in nine MECs. A total of 852 prokaryotic draft genomes representing 278 species, and 1476 phage contigs representing 873 phage species were assembled. Among high quality prokaryotic genomes (>95% completion), 55% carried a prophage, and the three *Desulfobacterota* spp. that dominated the anode communities all carried prophages. *Geobacter anodireducens*, one of the bacteria dominating the anode communities, carried a CRISPR spacer showing evidence of a previous infection by a *Peduoviridae* phage present in the liquid of some MECs. *Methanobacteriaceae* spp. and an *Acetobacterium* sp., which dominated the cathodes, had several associations with *Straboviridae* spp. The results of this study show that phage communities in MECs are diverse and interact with functional microorganisms on both the anode and cathode.

## Introduction

The ability of microorganisms to exchange electrons with solid minerals and metals via extracellular electron transfer appears to be widespread in nature [1, 2]. Extracellular electron transfer can also take place between cells, such as in co-cultures of *Geobacter* spp. [3, 4], and by cable bacteria, which are capable of centimeter-scale electron transfer across the anaerobic/aerobic interface of sediments [5, 6]. The ability of microorganisms to carry out extracellular electron transfer has been utilized in microbial electrochemical systems to catalyze conversions between chemical energy and electrical energy. One type of system is the microbial electrolysis cell (MEC), which can be used to produce energy carriers such as hydrogen- and methane gas with electrical power and dissolved organic compounds as input [7, 8]. In an MEC, a microbial community on the anode oxidizes organic compounds and generates electrical current. If the system is fed with wastewater, the anodic oxidation can contribute to wastewater treatment [9]. An external power source providing a small voltage input enables the electrical current to flow to the cathode where hydrogen ions are reduced to hydrogen gas or carbon dioxide is reduced to methane or other organic compounds. Although the hydrogen gas evolution reaction can occur abiotically, the cathode reactions may also be catalyzed by a microbial community [10, 11]. The anode communities contain electrogenic bacteria such as *Geobacter* spp, which are known for their ability to generate electric current by transferring electrons to a solid electrode [12]. The microbial community found on the cathode generally consists of methanogens, such as *Methanobacterium* spp. known to produce CH_4_ by catalyzing the reduction of CO_2_ [13]. *Acetobacterium* spp. that are involved in the reduction of CO_2_ to acetate are also commonly found in the cathode community [14–16].

The function of microbial electrolysis cells (MECs) is highly dependent on the microbial community in the system. The bacterial and archaeal community composition has been investigated in several studies, but nothing is known about the viral community. Bacteriophages, also known as “phages”, are viruses that specifically target, infect, and replicate within bacteria and archaea. Phages are typically either virulent or temperate. Virulent phages undergo the lytic cycle in which the phage infects its host and takes over its replicative machinery. The phage then uses the host to replicate the virion and synthesize the necessary viral proteins. Once the assembly of new virions is complete, the host cell undergoes lysis resulting in the release of virions that can continue to infect new host cells [17]. In contrast, temperate phages can undergo the lysogenic cycle in which viral DNA will be incorporated into the host genome resulting in prophages [18]. As the host replicates, the prophages are also replicated creating new cells (i.e. lysogens) containing the viral genome. If the host condition deteriorates due to stressors, such as nutrient depletion or UV light, the dormant prophage will undergo induction resulting in activation of the lytic cycle [17, 19]. Studies have shown that phages modulate the microbial community in various environments [20, 21]. For instance, a study of a coastal marine ecosystem suggested that phages preserved microbial diversity and richness [22]. Another study showed that a prophage could impact the bacterial colonization of mammalian guts by providing an advantage to its host in competition with strains lacking the prophage [23]. Regulation of the microbial community by phages has also been observed in full-scale anaerobic digesters and bioreactors treating industrial wastewater [24, 25]. The phages can regulate the abundance and diversity of microbial species in the systems [26, 27] and affect the metabolism and phenotype of the host; e.g. phages have been observed to promote biofilm formation in *Shewanella oneidensis* [28].

Although the bacteria and archaea responsible for the anode- and cathode functions of MECs have been identified in several studies, their interactions with phages have not been studied. The aim of this study was, therefore, to identify phages present in MECs and assess possible interactions with prokaryotes in the systems. Metagenomic sequencing of electrochemically active biofilms and virus-like particles (VLP) was carried out in multiple single-chamber MECs.

## Materials and methods

### Experimental setup

The experimental setup and performance of the MECs was previously described in Abadikhah et al [29]. Briefly, nine single-chamber MECs were operated for 104 days. The MECs had carbon cloth anodes and either steel, titanium, or carbon nanoparticle covered carbon paper as cathodes. Nutrient medium made in accordance with Saheb-Alam et al [16] supplemented with trace mineral solution [30] and organic carbon (0.6 g/L sodium acetate, 0.4 g/L sodium propionate and 0.32 g/L sodium butyrate), was added to the systems resulting in a total volume of 70 ml in each MEC. Each MEC was inoculated with 5 ml of mesophilic anaerobic digester sludge. Every 2–3 days, 50 ml of the medium was replaced with fresh nutrient medium. The cell voltage was kept at a 1 V using a multichannel potentiostat (Palmsense). The MECs with carbon nanoparticle cathodes were named C1, C2, and C3; those with titanium cathodes were named T1, T2, and T3; and those with steel cathodes were named S1, S2, and S3 (Fig. S1, supplementary material).

### VLP concentrations

Concentrations and size distribution of VLP in the liquid of the MECs were measured on four occasions during the first 30 days of the experiment using nanoparticle tracking analysis (NanoSight NS300, Malvern), as described in Modin et al. [31].

### Sampling and DNA extraction

Samples of the prokaryotic community were taken from biofilms on the anode- and cathode surfaces and the tubing walls, and suspended sludge at the end of the experimental run (Fig. S1). A sample was also taken from the inoculum at the start of the experiment and from foam generated due to excessive gas generation during a malfunctioning electrochemical test in MEC S3 on Day 60. The biofilms on the carbon cloth anodes and carbon nanoparticle covered carbon paper cathodes were sampled using a sterilized scissor to cut the material into smaller fragments, while the titanium and steel cathodes were harvested using a sterile spoon to scrape the biofilm from the surface of the material. The DNA was extracted using the FastDNA Spin kit for Soil (MP Biomedicals). The DNA extraction was performed in accordance with the manufacturer’s instructions except for the homogenization, which was done twice.

Samples targeting the active phage community were taken from the liquid in the system at the end of the experiment. Up-concentration of phage particles (i.e. VLP), DNA extraction, library preparation, and sequencing were carried out as in Modin et al [31]. Briefly, the samples were filtered through 0.2 μm polyethylene sulfone filters to remove bacteria. The remaining VLP were concentrated using cellulose membrane filters with a 100 kDa molecular weight cutoff (Amicon Ultra-15, Millipore). Extracellular DNA was removed by treatment with DNase I (20 U, Invitrogen) and phage DNA was extracted using Norgen’s Phage DNA Isolation Kit (Norgen Biotek). DNA sequencing was done using a NovaSeq 6000 system (Illumina).

### Bioinformatics

The DNA sequencing resulted in two datasets: the prokaryotic and the VLP. The raw sequence reads from both datasets were quality filtered using fastp v0.23.2 [32] and normalized to a target depth of 100 and a minimum depth of 2 using BBNorm (https://sourceforge.net/projects/bbmap/). The prokaryotic sequences were assembled into contigs using megahit v1.2.9 [33] with the presets meta-large. Contigs at least 2000 bp in length were binned sample-by-sample using both Vamb v3.0.2 [34] and Metabat v2.12.1 [35], and DAS Tool v1.1.4 [36] was used to select consensus bins. The bins were grouped into species clusters using dRep v3.3.0 [37], which uses CheckM [38] for estimating completeness and contamination of the bins. An average nucleotide identities (ANI) threshold of 95% was used to define species clusters and dRep identifies one of the bins as species representative based on genome quality and length. Species clusters containing at least one representative with >70% completeness and < 10% contamination were retained for further analysis. The taxonomic affiliation of each species representative was determined using GTDB-TK v2.1.1 with the GTDB database release207_v2 [39, 40]. The relative abundances of the species in each sample were estimated using CoverM v0.6.1 (https://github.com/wwood/CoverM) with bwa-mem v0.7.17 as mapper [41]. Phage sequences within the prokaryotic bins were identified using VIBRANT [42] and PhageBoost [43]. CheckV [44] was used to assess the quality of the identified phage sequences and those having a length of at least 5 kb and containing at least one viral gene were kept for further analysis.

The VLP sequences were assembled into contigs sample-by-sample using metaviralspades v3.15.3 [45]. The contigs were checked with CheckV and VIBRANT. Contigs that were classified as viral by VIBRANT, had at least one viral gene identified by CheckV, and were at least 5 kb in length were kept for further analysis.

The phage sequences identified in the prokaryotic dataset and the phage contigs in the VLP dataset were combined. PhaTYP [46] was used to determine lifestyle (virulent or temperate). Phage species clusters were generated using dRep at an ANI of 95%. In each cluster, the contig with the highest completeness according to CheckV was selected as species representative and CoverM was used to calculate the relative abundance of each phage species in each sample. Taxonomic classification was done using PhaGCN2 [47].

If a phage sequence was detected in a prokaryotic bin and was identified as temperate by PhaTYP, VIBRANT, or PhageBoost, it was classified as a prophage of the prokaryote. If the prophage sequence was also detected among the phage sequences assembled from the VLP dataset, this would indicate induction of the prophage in the MECs. If a CRISPR spacer found in a bin in the prokaryotic dataset matched a phage sequence, that would indicate a previous infection of that prokaryotic species by the phage. CRISPR spacers were identified using MinCED v0.4.2 [48] and matched to the phage sequences using SpacePHARER v5.c2e680a [49].

Differences in microbial community composition between samples were calculated using the Hill-based framework for beta diversity [50] using qdiv [51]. The Hill-based framework makes it possible to systematically assess the impact of relative abundance on the dissimilarity by varying the diversity order. Principal coordinate analysis (PCoA) was also done using qdiv.

## Results

### Prokaryotes

**Figure 1:**
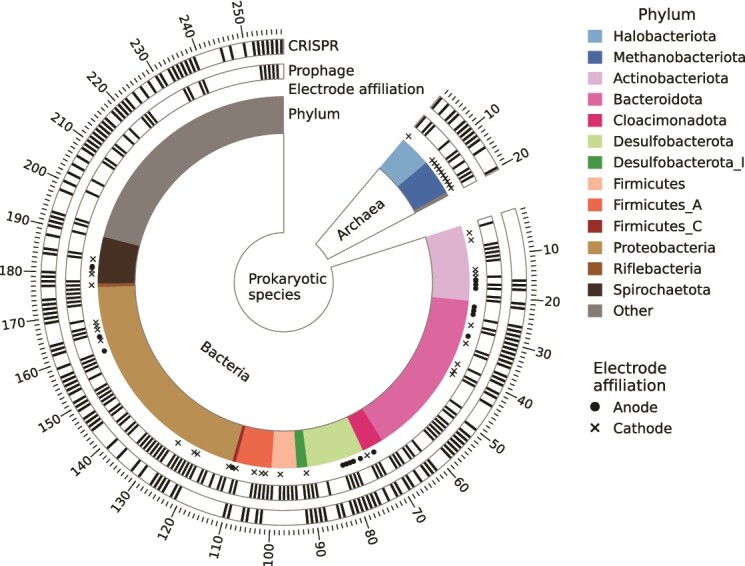
The 278 prokaryotic species. Black lines indicate species that contained a prophage or a CRISPR region. The colored bars show phyla containing electrode-affiliated species, which are indicated by • (anode) or x (cathode). Phyla without an electrode-affiliated species are grouped in the category called “other”, which include Acidobacteriota, Armatimonadota, Atribacterota, bacteria; KSB1, bacteria; UBA3054, Bdellovibrionota, Caldisericota, Campylobacterota, Chlamydiota, Chloroflexota, cyanobacteria, Desulfobacterota_G, Elusimicrobiota, Eremiobacterota, Fermentibacterota, Firmicutes_B, Firmicutes_G, Gemmatimonadota, Hydrogenedentota, Margulisbacteria, Methanobacteriota_B, Myxococcota, Omnitrophota, Patescibacteria, Planctomycetota, Synergistota, Thermotogota, and Verrucomicrobiota.

Sequencing of the 26 samples of the prokaryotic community resulted in a total of 330 gigabases of which 96.5% were retained after quality filtering. Assembly and binning resulted in 852 bins with completeness of 50–100% and contamination of 0–10% according to CheckM. The bins were dereplicated into 278 prokaryotic species, which included 20 archaea and 258 bacteria (Fig. 1). Each species was represented by the highest quality bin, as determined by dRep (Supplementary file, Table S1). Although *Proteobacteria* (*Pseudomonadota*) was the phylum with the highest number of genome bins, the anode and cathode samples were dominated by *Desulfobacterota* spp. and *Methanobacteriota* spp., respectively. The prokaryotes in suspension were diverse with methanogens within *Halobacteriota* and bacteria within *Bacteroidota* as major groups. The tubing samples contained *Proteobacteria*, *Bacteroidota*, and *Actinobacteriota* spp. The mesophilic anaerobic digester sludge used as inoculum contained mainly fermentative bacteria within *Fermentibacterota* and methanogens within *Halobacteriota* (Fig. S2, supplementary material).

**Figure 2:**
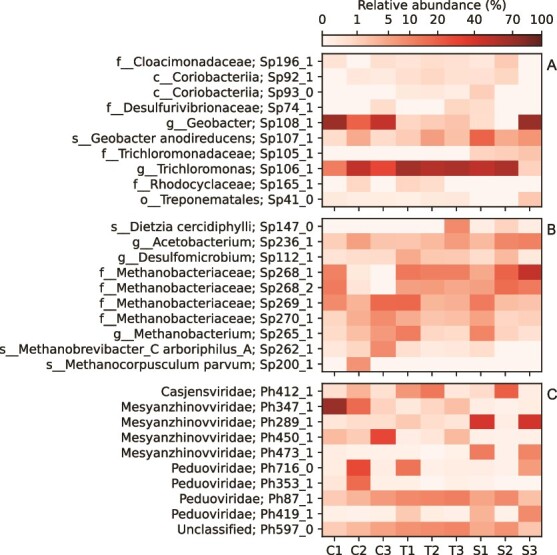
Heatmap of the most abundant prokaryotic species on the anode (a) and the cathode (b), and the most abundant phage species suspended in the liquid of the MECs (c). Taxonomic classifications are shown together with our identification code for the prokaryote or phage species.

In MECs, the prokaryotes affiliated with the electrode surfaces are particularly important for the function of the systems as they may be electrochemically active or directly interact with electroactive species. A species was deemed to be putatively electrode-affiliated if it had a mean relative abundance over 0.1% in either the anode or cathode samples, and a mean and maximum relative abundance in the anode- and cathode samples exceeding its mean and maximum relative abundance in tubing, suspension, and inoculum samples. This resulted in 17 species being identified as anode-affiliated and 40 species as cathode-affiliated. The electrode-affiliated species were spread across multiple phyla (Fig. 1). The most abundant electrode-affiliated species are shown in Fig. 2a-b. In the anode biofilms, a *Trichloromonas* sp. (Sp106_1) was most abundant in six out of nine samples and a *Geobacter* sp. (Sp108_1) was most abundant in the other three. *Geobacter anodireducens* (Sp107_1) also had high relative abundance (Fig. 2a). Among the 10 most abundant cathode-affiliated prokaryotes, seven were methanogens. Particularly *Methanobacteriaceae* spp. were abundant. An *Acetobacterium* sp. (Sp236_1) was also detected in all cathode biofilms (Fig. 2b).

### Distribution of prophages in prokaryotic genomes

**Figure 3:**
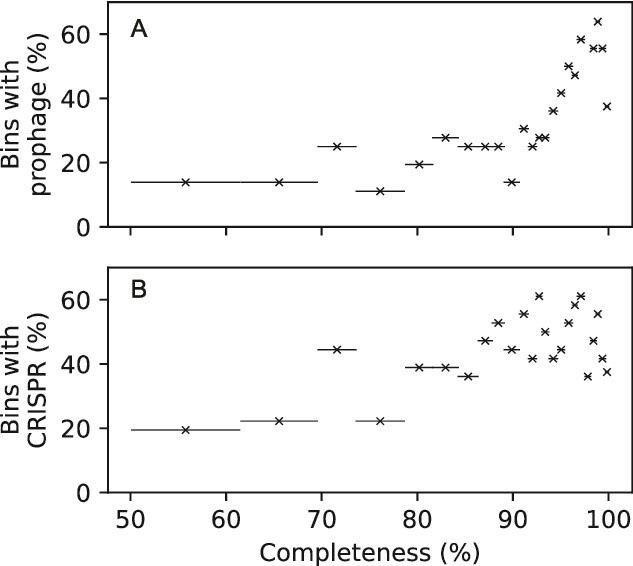
The proportion of bins with prophage (a) and CRISPR region (b) for group of bins with varying level of completeness (assessed by CheckM). To calculate the fractions, the bins were arranged into groups of 36 based on increasing completeness values. The horizontal bars show the completeness range for the group.

Prophages could be detected in 294 out of the 852 bins (~35%), which corresponded to 141 of the 278 species (~51%). In Fig. 1, the species is marked as containing a prophage if it was detected in at least one of the bins in the species cluster. In many cases, a species was represented by several bins assembled from different samples. In the entire data set, 90 species were represented by multiple bins and had a prophage detected in at least one of the bins. In 72 of these, the prophage could not be detected in all bins of the species and in 31 cases, the bin chosen a species representative did not contain the prophage. In general, the likelihood of finding a prophage in a bin increased with completeness. For bins with over 95% completeness, 55% had at least one prophage. Among bins with <70% completeness, only 16% had a prophage (Fig. 3a). Among the 17 anode-affiliated species and 40 cathode-affiliated species, a bin with a prophage could be detected in eight and 26 species, respectively.

### Distribution of CRISPR in prokaryotic genomes

CRISPR help prokaryotes defend themselves against foreign DNA such as plasmids and phages. Detection of CRISPR can, thus, be evidence of past phage infections [52, 53]. CRISPR regions could be detected in 374 of the 852 bins (~44%), which corresponded to 154 out of the 278 species (~55%). In Fig. 1, the species is marked as containing a CRISPR region if it was detected in at least one of the bins in the species cluster. In total, 95 species were represented by multiple bins and had a CRISPR region detected in at least one of the bins. In 59 of these, CRISPR regions could not be detected in all bins of the species and in 24 cases, the bin chosen a species representative did not contain a CRISPR region. As in the case of prophages, the likelihood of finding a CRISPR region in a bin increased with completeness (Fig. 3b). For bins with over 95% completeness, 50% had CRISPR regions. Among bins with <70% completeness, only 24% had a CRISPR regions. Among the anode- and cathode-affiliated species, a bin with a CRISPR region could be detected in 12 and 24 species, respectively.

### Phage species

**Figure 4:**
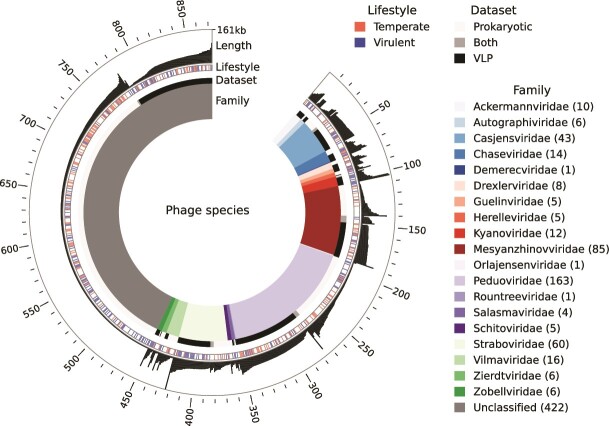
Overview of the 873 phage species. The inner colored ring shows family-level taxonomic classification. The number of different phage species in each family are shown as numbers within parentheses in the legend. The *dataset* bar shows if the phage species was detected in the VLP, prokaryotic, or both datasets. The colored *lifestyle* bar shows if the phage species is predicted as temperate or virulent. No marking means that the lifestyle prediction was ambiguous. The outer bar chart shows the length of each phage (the longest was 161 kb).

VLP concentrations in the nine MECs were measured during the initial phase of the experiment. It peaked at 7.0·10^10^(±2.1·10^10^) VLP/ml after 7 days of operation and decreased to 3.4·10^10^(±1.5·10^10^) VLP/ml on Day 30. Most of the VLP had a hydrodynamic diameter of ~120 nm (Fig. S3, supplementary material). Sequencing of VLP collected from the MECs at the end of the experimental run resulted in 61 gigabases of which 96.2% were retained after quality filtering. Together with the 815 prophages obtained from the prokaryotic bins, a total 1476 phage sequences were detected. Dereplication resulted in 873 phage species (Fig. 4, Supplementary file Table S2). Family-level taxonomic classification could be assigned to 459 phage species. All 22 detected phage families belonged to the *Caudoviricetes* class. The largest families were *Peduoviridae* (163 species), *Mesyanzhinovviridae* (85), *Straboviridae* (60), and *Casjensviridae* (43). Among the 10 most abundant phages found suspended in the liquid, four were *Mesyanzhinovviridae* and four were *Peduoviridae*. There was also one *Casjensviridae* and one unclassified phage. There was a large variation in distribution of phage families and species found among the VLP samples from the nine MEC (Fig. S4, supplementary material). Several of the most abundant phages were only detected in one or a few of the MECs (Fig. 2c).

In general, the phages assembled from the VLP dataset were longer than those from the prokaryotic dataset. The median phage length was 41.3 kb (36.6–49.2 kb, quartile 1–3) for the VLP sequences and 10.8 kb (7.2–19.1 kb) for the prokaryotic dataset. Among the 354 phage species detected in the VLP dataset, 126 were predicted as virulent, 76 as temperate, and 152 had an ambiguous prediction (i.e. VIBRANT and PhaTYP predictions did not agree). Among the 543 phage species detected in the prokaryotic dataset, 121 were predicted as virulent, 131 as temperate, and 291 had ambiguous prediction. Virulent phage sequences could have mistakenly been placed in prokaryotic genome bins by the binning software if they had similar kmer composition and relative abundance distribution across samples as the prokaryotic genome. The lifestyle prediction may also have been erroneous, e.g. temperate phages may have been classified as virulent by the software. For phage sequences assembled from the prokaryotic bins, the lifestyle prediction was related to the length of the phage contig. Phage sequences from prokaryotic genome bins that were predicted as virulent had a median length of 8.0 kb (6.3–11.3 kb, quartile 1–3) whereas those predicted as temperate had a median length of 16.0 kb (9.6–28.3 kb). The length was also a factor when it came to the taxonomic classification of the phage sequences. The unclassified phages had a median length of 11.4 kb (7.3–27.5 kb) and those with a taxonomic classification had a median of 38.5 kb (19.3–47.5 kb).

### Community differences between MECs

**Figure 5:**
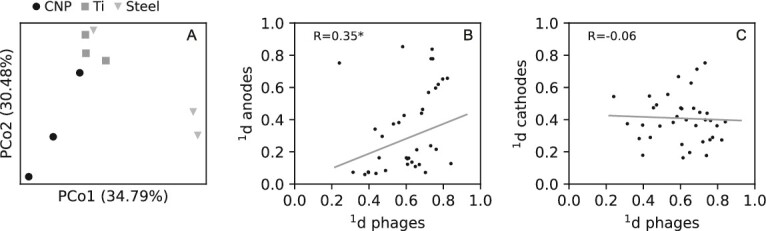
(a) PCoA of phage communities in the suspension (i.e. VLP) the nine MEC. (b-c) Correlation of pairwise dissimilarities between phage communities in suspension and prokaryotic communities on the either anodes (b) or cathode (s). Pearson’s correlation coefficient (R) shows the strength of the correlations. An asterisk (^*^) marks statistically significant correlation (*P <* 0.05). All dissimilarities were calculated at diversity order 1. Analyses based on diversity orders 0 and 2 are shown in Figs. S6-S7, supplementary material.

The prokaryotic communities differed mainly based on location within the MECs. The anode and cathode samples grouped into two different clusters in a PCoA (Fig. S5). The three cathode materials used in the nine MECs did not have any clear impact on neither the prokaryotic biofilms nor the phage communities suspended in the liquid (Fig. 5a).

To assess correlation between differences in phage communities and prokaryotic communities between MECs, Hill-based dissimilarity indices were used. These allow us to quantify the proportion of taxa that are different between pairs of samples and the diversity order (q) determines the emphasis put on the relative abundance of taxa. At q = 0, the index (^0^d) is the proportion of all detected taxa that are different; at q = 1 (^1^d), it is the proportion of taxa that can be considered common in the samples that are different; and at q = 2 (^2^d), it is the proportion of abundant taxa [50]. For the phage communities suspended in the liquid, the ^1^d index for pairs of MECs had a median value of 0.62 and a range of 0.36–0.80 (representing the 5^th^ and 95^th^ percentiles) suggesting that different MECs shared 38% (20–64%) of the common phage taxa (i.e. 1–^1^d) (Fig. 5b-c). The anodes shared 77% (21–93%) and the cathodes 61% (33–82%) of the common prokaryotic species. To explore whether MECs that had high dissimilarity in prokaryotic community composition also had high dissimilarity between phage community composition, pairwise dissimilarities for anode samples in different MECs were plotted against pairwise dissimilarities of phage communities (Fig. 5b) and the same analysis was done for cathode samples (Fig. 5c). In general, positive correlations were observed between anodes and phage communities with statistically significant correlation coefficients for diversity orders 0 and 1, but not 2 (Fig. 5b, Figs. S6-S7). The correlations between cathodes and phage communities were weak and not statistically significant (Fig. 5c, Figs. S6-S7).

### Phage-host associations

**Figure 6:**
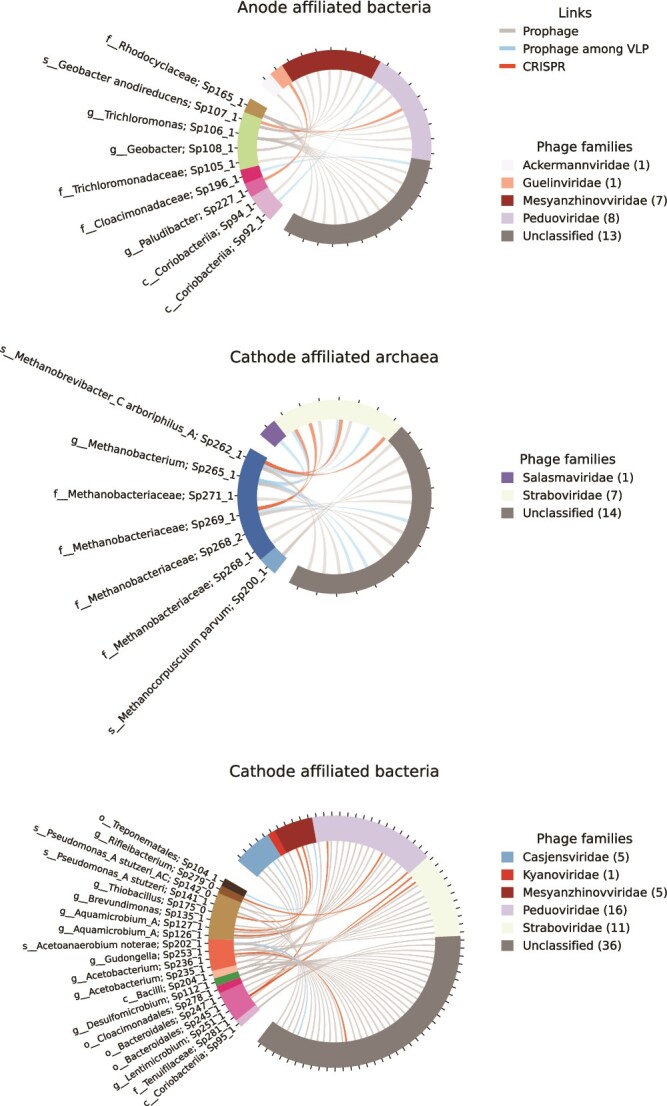
Associations between anode- and cathode affiliated prokaryotes and phage species. The type of association (prophage, prophage detected among the VLP in the liquid, and CRISPR spacer/protospacer match) are indicated with colored links. The numbers within parenthesis in the legends refer to the number of species associated with each phage family.

Associations could be established between 154 prokaryotes and 449 phage species. Figure 6 shows associations between electrode-affiliated prokaryotes and phages. For the anode-affiliated prokaryotes, nine were associated with 30 phages. Most of the associations were prophages found within the prokaryotic genomes. In two cases, the prophage could also be detected among the VLP in the liquid, which could mean that the prophage was being induced at the time of sampling. There were also two CRISPR-based matches with phages found among the VLP. Apart from associations with unclassified phages, most associations were with phages within *Mesyanzhinoviridae* and *Peduoviridae*. The three dominating *Desulfobacterota* spp. in the MECs (Sp106_1, Sp107_1, and Sp108_1) all carried prophages. *Geobacter anodireducens* (Sp107_1) also had a CRISPR-based match with a *Peduoviridae* phage. For the cathode-affiliated prokaryotes, 27 were associated with 96 phages. There were 86 prophages of which 10 could be found among the VLP in the liquid. There were also 12 CRISPR-based matches to phages among the VLP. For archaea, apart from associations with unclassified phages most associations were with *Straboviridae*. For bacteria, *Casjensviridae*, *Mesyanzhinovviridae*, *Peduoviridae*, and *Straboviridae* all had multiple phages represented. *Straboviridae*, which had many associations with cathode-affiliated prokaryotes, was primarily associated with an *Acetobacterium* sp. (Sp236_1) and two methanogens (Sp261_1 and Sp265_1) (Fig. 6). Among non-electrode-affiliated prokaryotes, 118 were associated with 326 phages. Apart from unclassified phages, most associations were with *Mesyanzhinovviridae* and *Peduoviridae* (Fig. S8).

The phages that could be associated with anode-affiliated prokaryotes made up 0.01–3.4% of the relative abundance of phage species found among the VLP suspended in the liquid of the MECs. The relative abundances for cathode-affiliated and non-electrode-affiliated prokaryotes were 0.26–3.7% and 5.1–20.7%, respectively. Phage species that could not be associated with any prokaryote in the systems made up 13–77% of the relative abundance.

## Discussion

Phages are known to be ubiquitous in nature [54] as well as in engineered systems such a bioreactors for wastewater treatment [55]. Here, we show that MECs are no exception, with measured VLP concentrations similar to those found in the anaerobic digesters and wastewater treatment plants [31, 56]. Approximately 55% of the prokaryotes in the MECs harbored prophages, which is similar to the 62% lysogens found in activated sludge used for municipal wastewater treatment [57]. The Piggyback-the-Winner model predicts that lysogeny is more prevalent in eutrophic environments with high microbial abundance [58]. A high microbial cell density and low diversity increases the likelihood that a cell becomes co-infected by two or more phages. Lysogens can be protected from these superinfections and may, thus, increase in abundance under such conditions [59]. These conditions favoring lysogeny can be found in MECs, which have high microbial abundance and relatively low diversity. In addition to a high fraction of lysogens, 50% of the prokaryotes had CRISPR regions, providing evidence of past phage infections. The presence of CRISPR-Cas systems is known to vary between phyla. *Desulfobacterota* and *Bacteroidota*, two phyla that were abundant in the MECs, were previously shown to have CRISPR-Cas prevalence of ~25% and 45%, respectively [60]. Prokaryotic draft genomes obtained through metagenomic sequencing are often fragmented and incomplete, which make accurate assessments of the prevalence of prophages and viral defense systems difficult. Although the fractions of 55% prokaryotic genomes with prophages and 50% with CRISPR regions were obtained for genome bins with >95% completeness, the values may be underestimations. The completeness of prokaryotic draft genomes is estimated based on marker genes [38], which may not necessarily reflect the ability of assembly and binning software to capture prophages and phage defense systems in the genomes. Thus, the actual proportion of prokaryotic species in the MECs with such components in their genomes may be even higher.

The phage species that could be taxonomically classified all belonged to the *Caudovirecetes* class, which contain double-stranded DNA viruses [61, 62]. The observation of DNA phages in this study is logical considering that the methods for nucleic acid extraction and sequencing focused on DNA, not RNA. Many of the taxonomically unclassified phages had short sequence length and were likely partial phage genomes. Longer sequences contain more information, which increases the likelihood of accurate taxonomic classification [47]. The composition of phages found among the VLP differed. *Mesyanzhinovviridae* was the most abundant family in four MECs, *Peduoviridae* was most abundant in four, and *Casjensviridae* was most abundant in one (Fig. S2). We hypothesized that differences in prokaroytic community composition could be correlated with differences in phage community composition in the MECs, but only weak positive correlations between anodes and VLP were observed, and no correlation was observed between cathodes and VLP (Fig. 5). Nevertheless, phage infections could be a factor that drives prokaryotic community dissimilarity, which may be interpreted as ecological drift when parallel reactor systems are analysed [e.g. [63, 64]].

Links could be established between electroactive prokaryotes and phages found among the VLP. CRISPR spacers indicating acquired immunity against phages in the system could be observed for both anode- and cathode-affiliated prokaryotes. Among the anode-affiliated taxa, a *Coriobacteriia* sp. (Sp92_1) and a *Cloacimonadaceae* sp. (Sp196_1) had prophages being found among the VLP, suggesting induction had taken place (Fig. 6). Induction of prophages in electrogens could lead to decreased current generation in MECs. A previous study with a pure culture of *Geobacter sulfurreducens* showed a reduced current generation when prophages were induced [65]. Among the cathode-affiliated methanogens, induced prophages could be observed for two *Methanobacteriaceae* spp. and one *Methanobrevibacter arboriphilus*. Methanogens are important microorganisms both in MECs and in anaerobic digestion. In MECs, they are unwanted if the target product is hydrogen gas, and in anaerobic digesters they are essential for methane production. Virulent tailed phages have previously been shown to target *Methanobacteriales* spp. [66, 67]. A more recent study investigated phages in enrichment cultures of methanogens within the genera *Methanobacterium* and *Methanobrevibacter*. A siphovirus-like phage within *Caudoviricetes* infecting *Methanobacterium* sp. was found. The phage did not belong to a known viral family and the authors suggested the name *Speroviridae* [68]. The methanogens on the cathodes in the MECs had several associations with *Straboviridae*, which formerly belonged to the morphology-based family Myoviridae [61]. A recent survey of methagenomes from methanogenic and anaerobic methane-oxidizing environments found Myoviridae to be more prevalent than Siphoviridae and Podoviridae, which are two other morphology-based families of tailed phages that were recently abolished in phage taxonomy [61, 69].

Prophage induction in MECs may be related to variations in environmental conditions. When MECs are fed in batch cycles, as they were in this study, the microbial communities are exposed to high substrate availability at the beginning of the batch cycle and low at the end. Variations in substrate could cause induction of prophages [70, 71]. Another factor that varies is the local pH near the electrode surfaces. At the cathode, current generation leads to consumption of hydrogen ions for production of hydrogen gas, which increases pH. The opposite occurs at the anode where hydrogen ions are liberated during the oxidation of organics. Changes in pH has been shown to induce prophages in certain bacteria [72, 73]. Host density is another factor affecting prophage induction and it has been noted that temperate phages switch from lytic activity to lysogeny as a survival mechanism when availability of bacterial host are low [74]. Some temperate phages have been shown to use signaling molecules to determine the abundance of hosts in its vicinity and whether to switch between lysogenic and lytic cycles [75–78]. In MECs, current density and the abundance of electrochemically active microorganisms on the electrode surfaces vary over time. We have previously observed that the current density increases rapidly during the enrichment of an electrogenic community on the anode. Then, the current density decreases somewhat and stabilizes at a lower level [29]. The phenomenon may be related to changes in biofilm thickness causing mass transfer limitation of the substrate. Prophage induction during the rapid increase in host density occurring during the enrichment of electrogens could also be an explanation for variations in current density in MECs. This study shows that phage interact with functional microorganisms on anodes and cathodes in MECs. Further research should examine temporal dynamics of the phage community and its relationship with MEC function.

## Supplementary Material

Supplementary_figures_ycae143

Supplementary_tables_ycae143

## Data Availability

The raw sequencing reads are available in the Sequence Read Archive (SRA) under project number PRJNA839919.
