# Supplementary material for: Viral diversity and host associations in microbial electrolysis cells
Source: ISME Commun. 2024 Nov 15;4(1):ycae143. doi: 10.1093/ismeco/ycae143 (PMC11629682; doi:10.1093/ismeco/ycae143)
Supplement: Supplementary_figures_ycae143 [file supplementary_figures_ycae143.pdf]

Supplementary material

Viral diversity in microbial electrolysis cells

Marie Abadikhah, Frank Persson, Anne Farewell, Britt-Marie Wilén, Oskar Modin

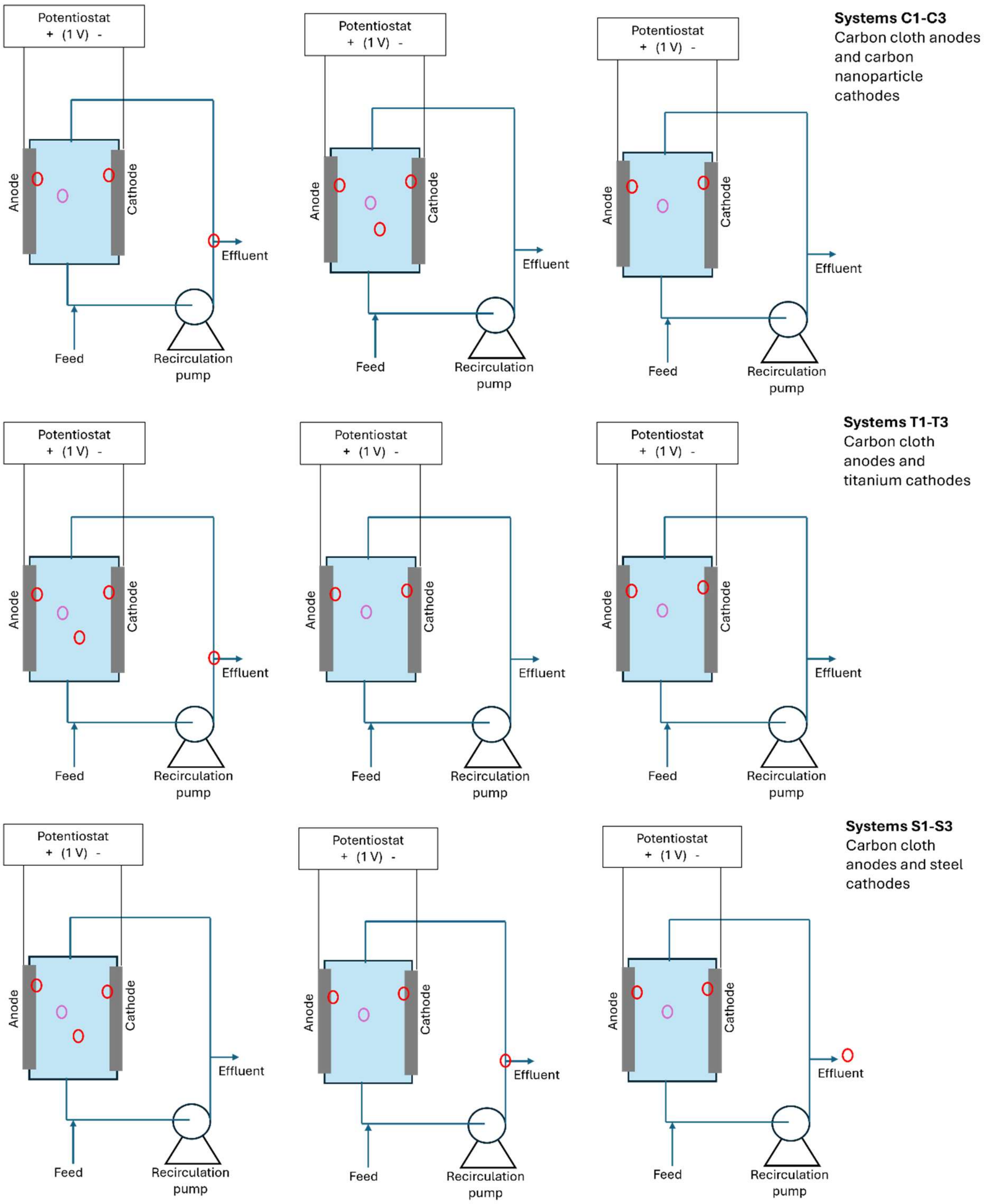

Fig. S1. Nine microbial electrolysis cells were operated in parallel. Sampling spots for prokaryotes are marked with red circles. Anodes and cathodes were samples in all MECs. Suspended sludge (C2, T1, S1), tubing walls (C1, T1, S2), and foam (S3) were only sampled in some of the MECs. Sampling spots for virus-like particles are marked with purple circles and was carried out in all MECs.

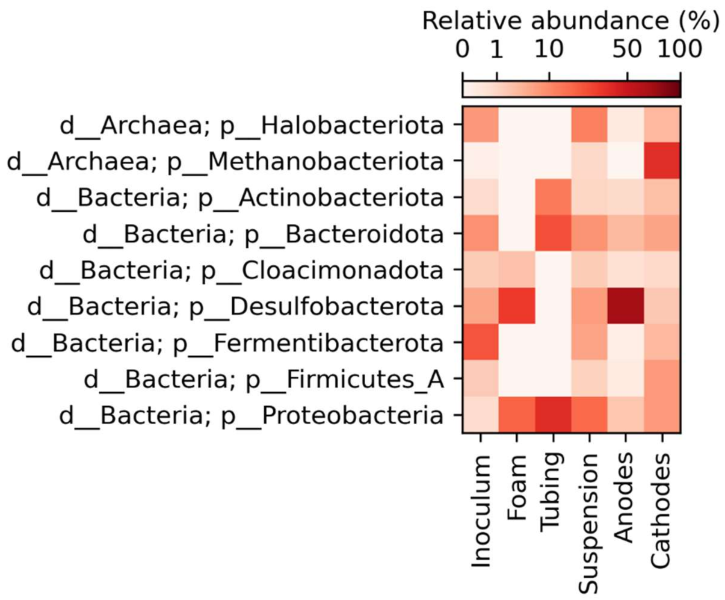

Fig. S2. The most abundant phyla in the different types of samples collected from the MECs.

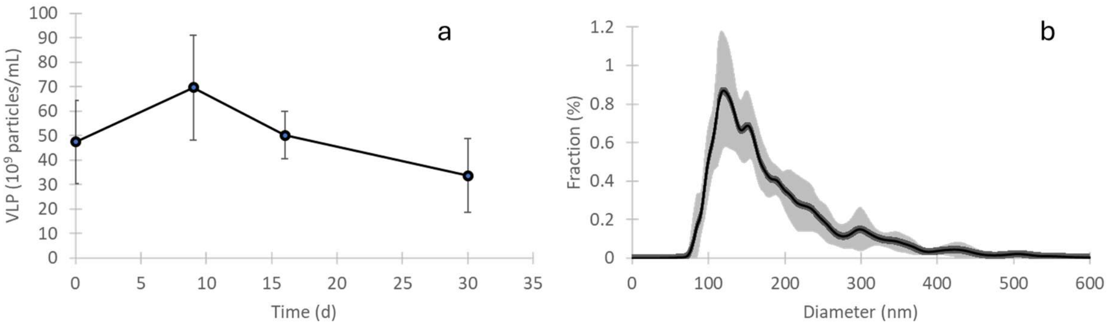

Fig. S3. (a) Concentration of virus-like particles (VLP) measured in the nine MECs (mean±standard deviation) during the initial phase of the experiment, which lasted 104 days. (b) Size distribution of the VLP measured as hydrodynamic diameter. Shaded region show standard deviation of measurements taken in the nine MECs.

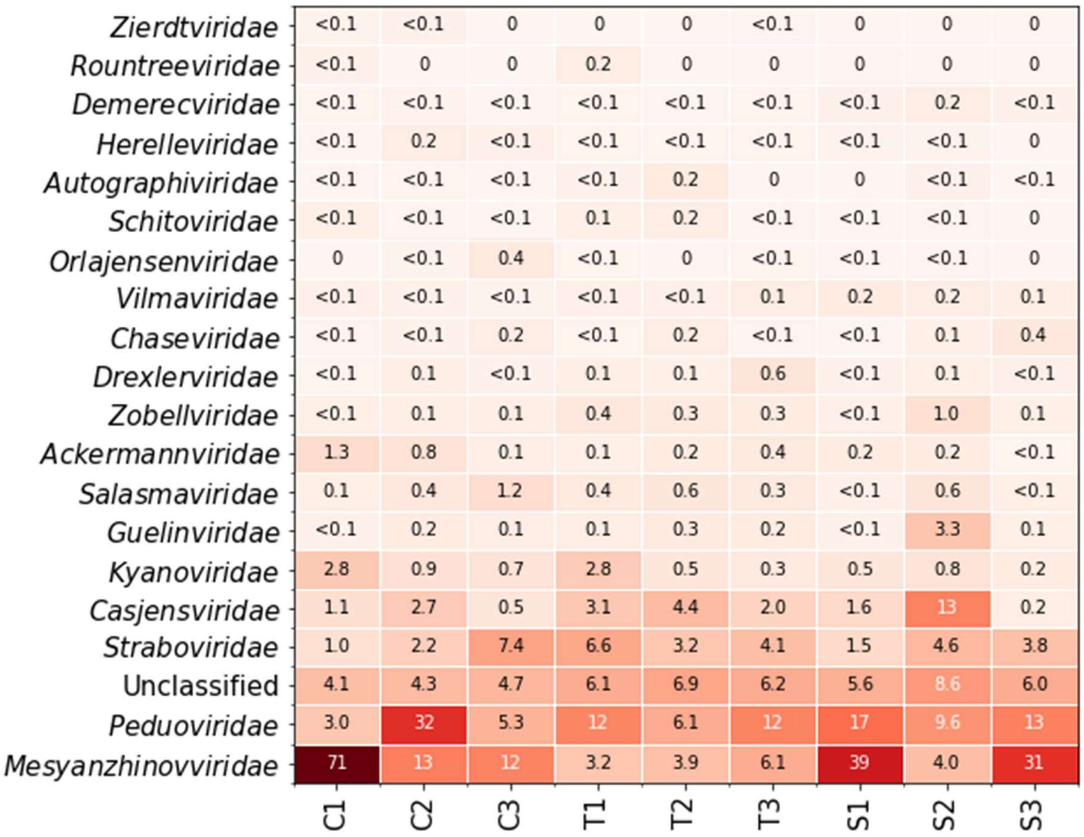

Fig. S4. The relative abundance of phage families among the sequence reads from virus-like particles in the nine microbial electrolysis cells.

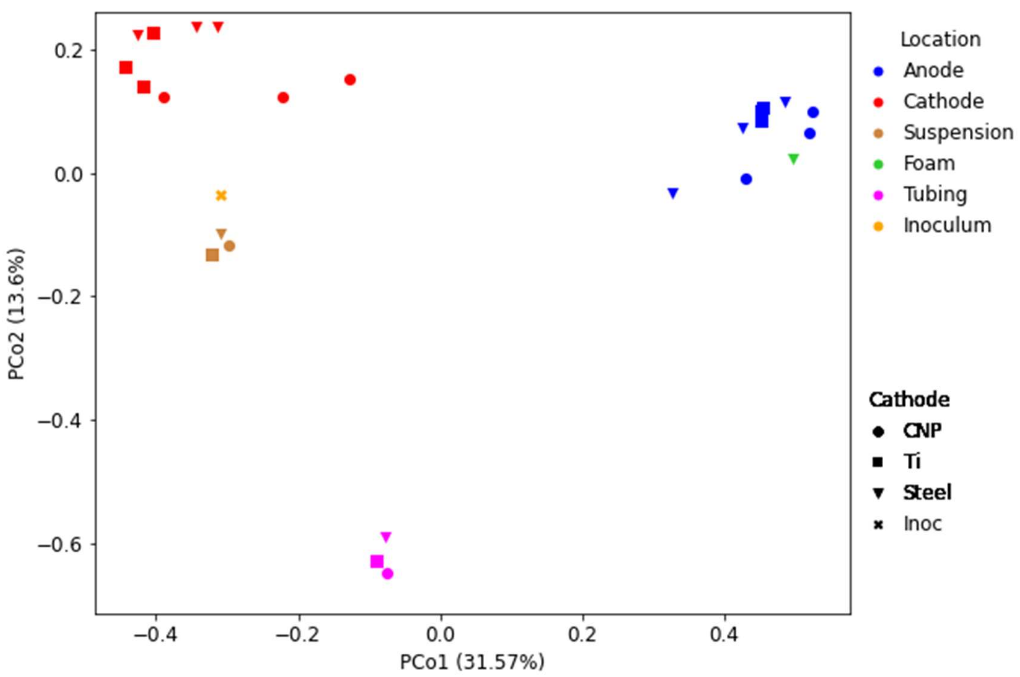

Fig. S5. Principal coordinate analysis (PCoA) based on the relative abundances of the prokaryotic bins in the samples. Hill-based dissimilarity index with diversity order 1 was used.

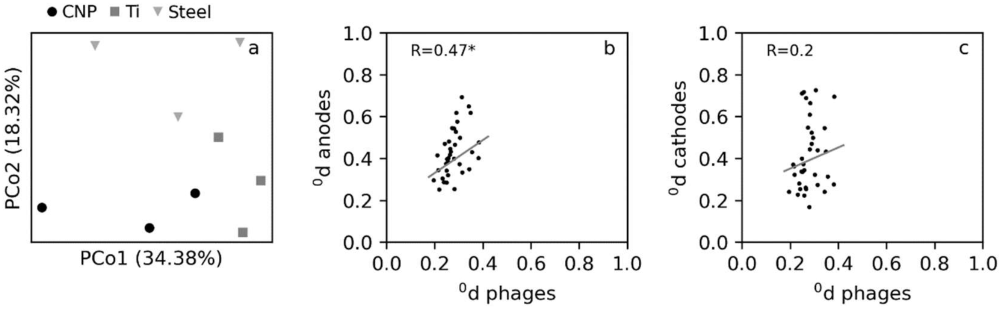

Fig. S6. (a) Principal coordinate analysis (PCoA) of phage communities in the suspension (i.e. VLP) the nine MEC. (b-c) Correlation of pairwise dissimilarities between phage communities in suspension and prokaryotic communities on the either anodes (b) or cathode (s). Pearson's correlation coefficient (R) shows the strength of the correlations. An asterisk (*) marks statistically significant correlation ($p<0.05$). All dissimilarities were calculated at diversity order 0.

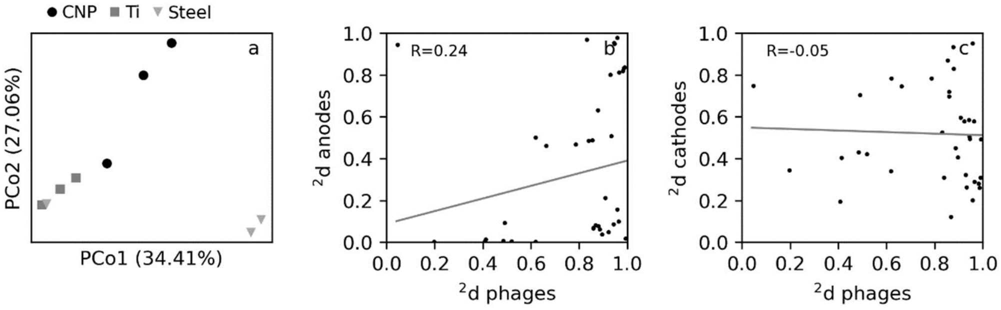

Fig. S7. (a) Principal coordinate analysis (PCoA) of phage communities in the suspension (i.e. VLP) the nine MEC. (b-c) Correlation of pairwise dissimilarities between phage communities in suspension and prokaryotic communities on the either anodes (b) or cathode (s). All dissimilarities were calculated at diversity order 2.

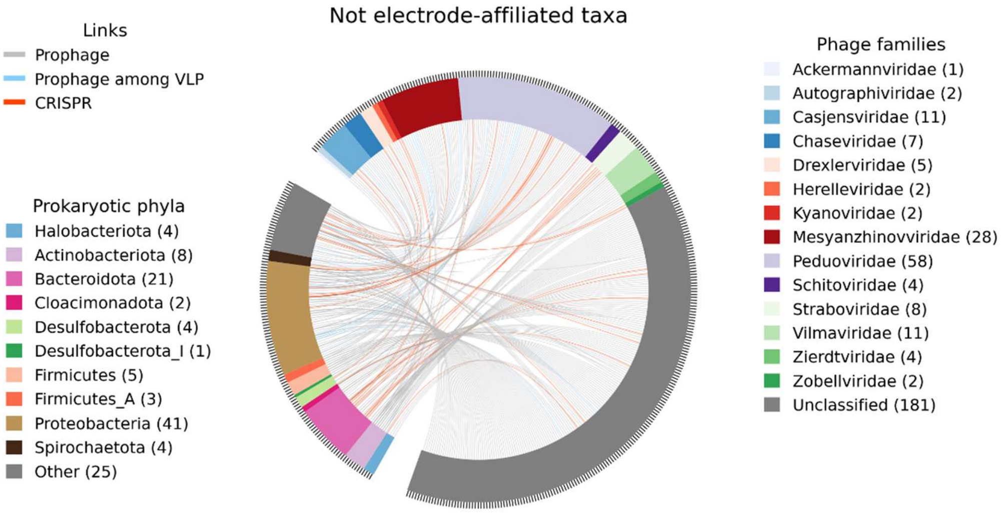

Fig. S8. Associations between non-electrode-affiliated prokaryotes and phage species. The type of association (prophage, prophage detected among the virus-like particles in the liquid, and CRISPR spacer/protospacer match) are indicated with coloured links. Among the prokaryotic phyla, the category "Other" includes Acidobacteriota, Armatimonadota, Bacteria, KSB1, Chlamydiota, Chloroflexota, Desulfobacterota_G, Elusimicrobiota, Firmicutes_B, Hydrogenedentota, Myxococcota, Thermotogota, and Verrucomicrobiota. The numbers within parenthesis in the legends refer to the number of species associated with each phage family or prokaryotic phylum.
